# Ginkgolide K attenuates neuronal injury after ischemic stroke by inhibiting mitochondrial fission and GSK-3β-dependent increases in mitochondrial membrane permeability

**DOI:** 10.18632/oncotarget.17967

**Published:** 2017-05-18

**Authors:** Xu Zhou, Hui-Ying Wang, Bin Wu, Cai-Yi Cheng, Wei Xiao, Zhen-Zhong Wang, Yu-Yu Yang, Ping Li, Hua Yang

**Affiliations:** ^1^ State Key Laboratory of Natural Medicines, China Pharmaceutical University, Nanjing, China; ^2^ State Key Laboratory of New-tech for Chinese Medicine Pharmaceutical Process, Jiangsu Kanion Pharmaceutical Co., Ltd., Lianyungang, China

**Keywords:** ginkgolide K, mitochondrial fission, mitochondrial permeability transition pore, neuron apoptosis, ischemic stroke

## Abstract

Ginkgolide K (GK) belongs to the ginkgolide family of natural compounds found in *Ginkgo biloba* leaves, which have been used for centuries to treat cerebrovascular and cardiovascular diseases. We evaluated the protective effects of GK against neuronal apoptosis by assessing its ability to sustain mitochondrial integrity and function. Co-immunoprecipitation showed that Drp1 binding to GSK-3β was increased after an oxygen-glucose deprivation/reperfusion (OGD/R) insult in cultured neuroblastoma cells. This induced Drp1 and GSK-3β translocation to mitochondria and mitochondrial dysfunction, which was attenuated by GK. GK also reduced mitochondrial fission by increasing Drp1 phosphorylation at Ser637 and inhibiting mitochondrial Drp1 recruitment. In addition, GK exposure induced GSK-3β phosphorylation at Ser9 and enhanced the interaction between adenine nucleotide translocator (ANT) and p-GSK-3β. This interaction suppressed the interaction between ANT and cyclophilin D (CypD), which inhibited mitochondrial permeability transition pore (mPTP) opening. Similarly, suppression of mitochondrial fission by Mdivi-1 also inhibited GSK-3β-induced mPTP opening. Treating mice with GK prevented GSK-3β and Drp1 translocation to mitochondria and attenuated mitochondrial dysfunction after middle cerebral artery occlusion. We therefore propose that by inhibiting mitochondrial fission and attenuating mPTP opening, GK exerts neuroprotective effects that mitigate or prevent neuronal damage secondary to ischemic stroke.

## INTRODUCTION

Acute ischemic stroke (AIS) is caused by a sudden interruption of blood supply to the brain and a leading cause of disability and mortality in adults worldwide. The brain's energy demands account for at least 20% of the total energy produced by the body [1]. Strokes alter brain energy balance and cause cerebral damage characterized by neuroinflammation, neuronal damage, and cerebral edema. Mitochondrial dysfunction, and the consequent impairment in cellular energy production, is at the center of the pathogenic changes caused by AIS.

Mitochondria are dynamic, double membrane-bound organelles that constantly undergo cycles of fusion and fission [2]. Dynamin-related protein 1 (Drp1) is a dynamin GTPase and its activation promotes mitochondrial fission [3]. Drp1 is largely dispersed in the cytosol, with only a fraction localizing to mitochondria under physiological conditions [4]. Drp1 translocates to puncta on mitochondria, where it promotes mitochondrial fission by coupling GTP hydrolysis with mitochondrial membrane constriction [5]. The activity of Drp1 can be modified in several ways, including sumoylation, ubiquitination, and phosphorylation [6]. Drp1 (Ser637) phosphorylation by cyclic adenosine monophosphate-dependent protein kinase (AMPK) attenuates its translocation to mitochondria and decreases mitochondrial fission [7]. After a stroke, ischemia/reperfusion (I/R) injury commonly results in mitochondrial fission and fragmentation, leading to neuronal apoptosis secondary to opening of the mitochondrial permeability transition pore (mPTP) [8].

The mPTP is comprised of the proteins Bax/Bak in the outer, and ANT, CypD, and phosphate carrier (PiC) in the inner, mitochondrial membranes [9]. Inner membrane pore opening leads to the release of low molecular weight compounds, mitochondrial membrane potential (MMP) collapse, and cristae remodeling [10]. The outer membrane pore opening results in the release of cytochrome *c* and activates the caspase cascade, leading to cell death [3]. It is now acknowledged that mitochondrial structure integrity disruption, reflected by reorganization of inner membrane cristae and opening of cristae junctions after mitochondrial fragmentation [11] is a key event in mPTP opening.

Glycogen synthase kinase-3β (GSK-3β), a serine/threonine protein kinase with crucial roles in mitochondrial homeostasis, is commonly involved in the pathogenesis of cardiac and cerebral diseases. The translocation of GSK-3β from cytosol to mitochondria promotes mPTP opening [12]. A previous study showed that phosphorylation of GSK-3β at Ser9 blocks its translocation, desensitizing mPTP opening during the reperfusion phase in myocardial I/R injury [13]. However, whether mitochondrial translocation of GSK-3β is associated with Drp1 recruitment is yet to be determined.

**Figure 1:**
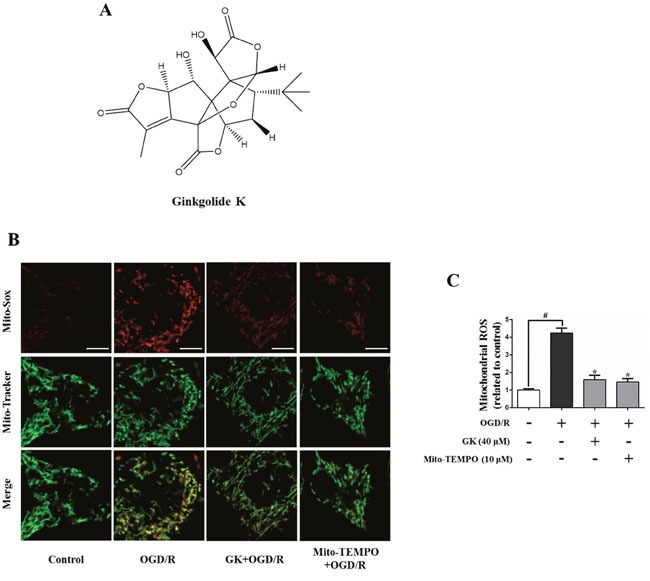
GK attenuates mitochondrial ROS generation after OGD/R After treatment with GK (40 μM) or Mito-TEMPO (10 μM) for 4h, N2a cells were exposed to OGD/R. **(A)** GK structure. **(B-C)** Mitochondrial-derived ROS was measured by Mito-Sox Red using confocal microscopy. Mito-Tracker green was used to locate mitochondria. Scale bar, 5 μm. Data are expressed as mean ± SD (n = 3). *p < 0.05 vs. OGD/R; #p < 0.01 vs. untreated control.

Ginkgolide K (GK), a natural product isolated from the leaves of *Ginkgo biloba* (Figure 1A), has been extensively used as a traditional medicine to ameliorate symptoms of cerebrovascular and cardiovascular diseases. Ginkgolides are natural antagonists of platelet-activating factor (PAF) receptor and protect neuronal function by reducing inflammation and oxidative stress after I/R injury. GK is a rare ginkgolide that has attracted much attention in recent years due to its potential in treating stroke and providing cardioprotection against endoplasmic reticulum stress injury following ischemia [14]. Previous research has confirmed that GK can cross the blood brain barrier in normal conditions [15]. In this study, we investigated the relationship between mitochondrial fission and fragmentation and mPTP opening in an *in vitro* model of I/R, and analyzed the roles of Drp1 and GSK-3β in the regulation of mPTP opening in a mouse model of stroke. Our findings suggest that inhibition of mitochondrial fission could be a potential strategy for AIS therapy.

## RESULTS

### GK reduces mitochondrial ROS production and suppresses mitochondrial fission in N2a cells

Mitochondria are the predominant source of reactive oxygen species (ROS) after tissue reperfusion, and inhibition of ROS production reduces mitochondrial oxidative damage [16]. To evaluate the ability of GK to prevent or attenuate ROS production, N2a cells were stained with the ROS indicator Mito-Sox and challenged with an oxygen glucose deprivation/reoxygenation (OGD/R) insult (an *in vitro* model of I/R injury). ROS production was increased after OGD/R, and this effect was attenuated by GK and Mito-TEMPO, a ROS scavenger (Figure 1B and 1C). These results suggest that GK can reduce oxidative stress in cultured neurons.

**Figure 2:**
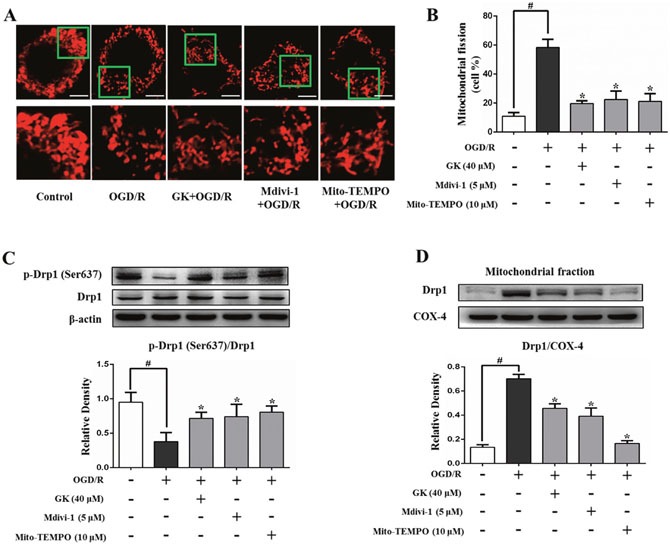
GK decreases mitochondrial fission after OGD/R N2a cells were treated with GK (40 μM), Mdivi-1 (5 μM), or Mito-TEMPO (10 μM) for 4h, prior to exposure to OGD/R. **(A-B)** Mitochondrial fission, represented by the percentage of fragmented mitochondria, was assessed by Mito-Tracker red fluorescence using confocal microscopy. Scale bar, 5 μm. **(C-D)** GK, Mdivi-1, and Mito-TEMPO treatment increased Drp1 phosphorylation at Ser637 and reduced Drp1 translocation to mitochondria after OGD/R. Data are expressed as mean ± SD (n = 3). *p < 0.05 vs. OGD/R; #p < 0.01 vs. untreated control.

As shown in Figure 2A and 2B, an increase in the proportion of fragmented mitochondria was evident after OGD/R (58.3 ± 5.7%), and this effect was attenuated in cells pre-treated with GK (19.7 ± 1.9%), Mdivi-1, a mitochondrial fission inhibitor (22.5 ± 5.8%), or Mito-TEMPO (21.1 ± 5.5%). Drp1 phosphorylation (Ser637) was markedly decreased in N2a cells exposed to OGD/R, an effect partially attenuated in cells previously exposed to GK, Mdivi-1, or Mito-TEMPO (Figure 2C), in which reduced Drp1 translocation to mitochondria was observed as well (Figure 2D). These results suggest that GK attenuates mitochondrial fission by reducing ROS production and preventing Drp1 localization to mitochondria following an ischemic/hypoxic insult.

### Mitochondrial fission mediates mPTP opening in a GSK-3β-dependent manner

**Figure 3:**
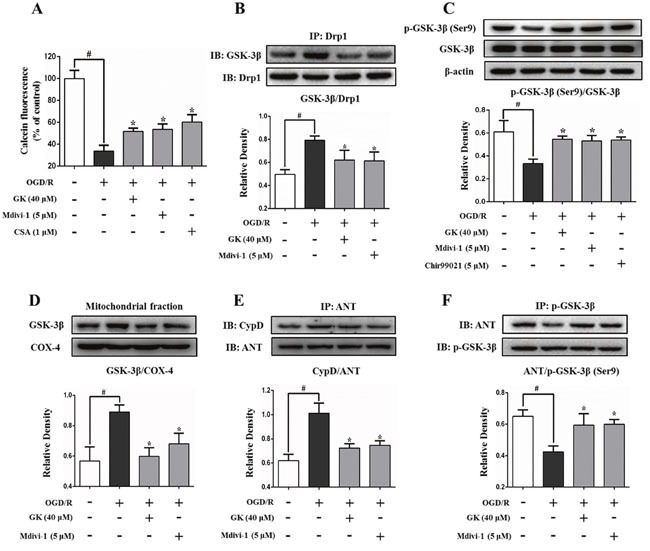
GK attenuates GSK-3β-associated mPTP opening After treatment with GK (40 μM), Mdivi-1 (5 μM), CSA (1 μM), or Chir99021 (5 μM) for 4h, N2a cells were exposed to OGD/R. **(A)** mPTP opening was increased after OGD/R. GK, Mdivi-1, and CSA treatment all attenuated the opening of mPTP. **(B)** Immunoprecipitation experiments show that the binding of Drp1 to GSK-3β was increased after OGD/R, and Mdivi-1 treatment reversed this effect. **(C)** GK, Mdivi-1, and Chir99021 promoted phosphorylation of GSK-3β at Ser9 after OGD/R. **(D)** GK and Mdivi-1 decreased GSK-3β in mitochondria. **(E-F)** GK and Mdivi-1 reduced the affinity of ANT toward CypD by increasing the binding of phospho-GSK-3β and ANT after OGD/R. Data are expressed as mean ± SD (n = 3). *p < 0.05 vs. OGD/R; #p < 0.01 vs. untreated control.

Mitochondrial fragmentation is a prerequisite for the opening of the mPTP [3]. We evaluated OGD/R-induced mPTP opening by measuring calcein-AM fluorescence intensity in cultured N2a cells. Results showed that the intensity of calcein-AM was significantly decreased (by 66.3%) after OGD/R, indicating an increase in mPTP opening (Figure 3A). However, the fluorescence intensity decay was partly prevented, by 18.1%, 19.9%, and 26.6%, respectively, in cells pre-exposed to GK, Mdivi-1, or cyclosporin A (CSA) (Figure 3A). We also observed that the binding of Drp1 to GSK-3β, which was increased after OGD/R, was attenuated by GK treatment (Figure 3B). GSK-3β translocation to mitochondria is an essential factor in mPTP opening and is kinase activity-dependent [12, 17]. Phosphorylation of GSK-3β at its Ser9 decreased after OGD/R, and this decrease was prevented by GK, Mdivi-1, and the GSK-3β inhibitor Chir99021 (Figure 3C). Consistent with this observation, GSK-3β expression in mitochondria was increased after the OGD/R insult, an effect also suppressed by GK or Mdivi-1 exposure (Figure 3D).

The mPTP comprises two main components, ANT and CypD in the inner mitochondrial membrane, that interact to promote mPTP opening [9]. As shown in Figure 3E, generation of the ANT/CypD complex increased significantly after OGD/R, while GK or Mdivi-1 pre-treatment decreased the binding of ANT to CypD by promoting the interaction of p-GSK-3β and ANT (Figure 3F). These results suggest that mitochondrial fission increases the binding of Drp1 to GSK-3β, and inhibition of mitochondrial fission reduces mPTP opening in a GSK-3β-dependent manner.

### GK alleviates mitochondrial dysfunction in N2a cells

**Figure 4:**
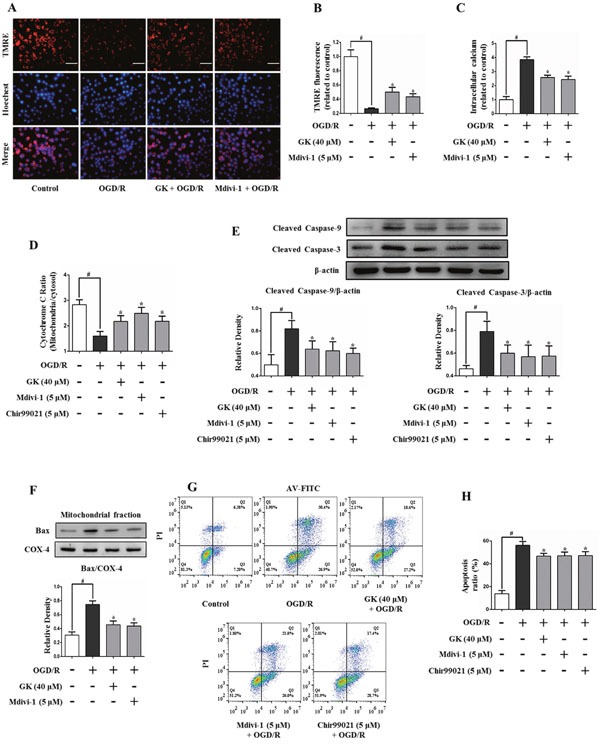
GK prevents mitochondrial dysfunction and apoptosis after OGD/R N2a cells were treated with GK (40 μM), Mdivi-1 (5 μM), or Chir99021 (5 μM) for 4h prior to OGD/R. **(A-B)** Mitochondrial membrane potential (MMP) was measured by TMRE labeling. MMP collapsed after OGD/R injury, and GK and Mdivi-1 pre-treatment attenuated this effect. Scale bar, 50 μm. **(C)** Intracellular calcium increased after the OGD/R insult, and this increase was reduced in GK- and Mdivi-1-treated cells. **(D)** Mitochondrial cytochrome *c* release (quantified by ELISA) was attenuated by GK, Mdivi-1, and Chir99021 pre-treatment in cells challenged with OGD/R. **(E-F)** The activation of caspases 3 and 9, as well as the expression of Bax in mitochondria, were attenuated by GK exposure prior to OGD/R. **(G-H)** OGD/R-induced apoptosis of N2a cells was reduced by previous exposure to GK, Mdivi-1, and Chir99021. The apoptosis assay was carried by AnnexinV-FITC/PI staining and flow cytometry. Data are expressed as mean ± SD (n = 3). *p < 0.05 vs. OGD/R; #p < 0.01 vs. untreated control.

Mitochondrial dysfunction subsequent to mPTP opening is characterized by MMP collapse, mitochondrial calcium overload, and cytochrome *c* release. To assess whether GK is protective against OGD/R-induced MMP collapse, N2a cells were stained with TMRE, a potentiometric fluorescent dye. A significant decrease in TMRE signal was observed in cells exposed to OGD/R (26.6 ± 1.3% vs 100 ± 9.6% in the control group). However, a significant attenuation in MMP loss (50.1 ± 6.5% and 43.3 ± 4.3%) was observed, respectively, in cells treated with GK or Mdivi-1 (Figure 4A and 4B). Furthermore, intracellular calcium levels were increased after OGD/R, and this effect was reduced upon treatment with GK (Figure 4C). In addition, the release of cytochrome *c* from mitochondria to the cytosol was also attenuated by GK, Mdivi-1, and Chir99021 (Figure 4D).

Apoptosis markers were next studied using western blot. Results showed that the OGD/R challenge induced an increase in the expression of cleaved caspase-9 and cleaved caspase-3, which was attenuated by pre-treatment with GK or Mdivi-1 (Figure 4E). Besides, mitochondrial Bax expression was significantly increased after OGD/R, denoting the opening of the mPTP [18], and GK treatment reversed this effect (Figure 4F). Annexin V-FITC/PI staining was further applied to measure cell apoptosis by flow cytometry. Apoptosis caused by OGD/R in N2a cells was attenuated by GK, Mdivi-1, and Chir99021 treatment (Figure 4G and 4H). These data indicate that GK can counteracted mitochondrial dysfunction and apoptosis after an OGD/R insult.

### GK ameliorates cerebral injury after I/R

**Figure 5:**
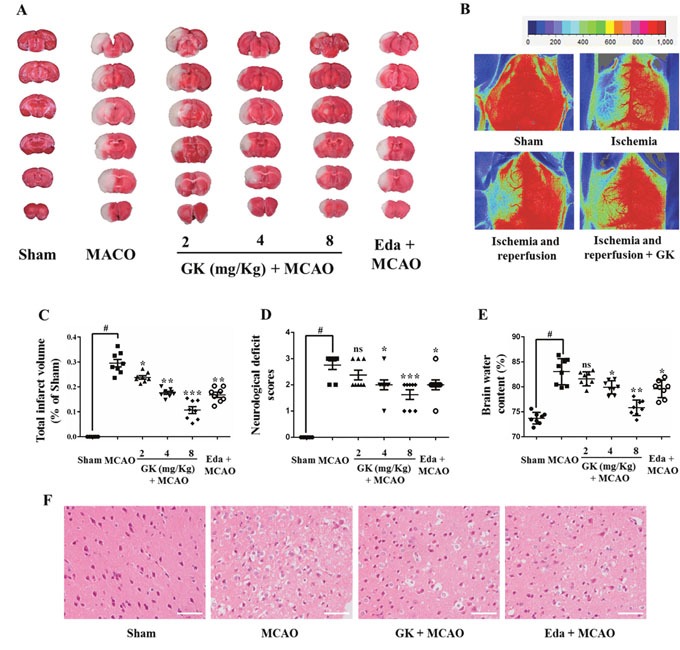
GK ameliorates I/R-induced brain injury **(A)** The infarct volume of mouse brains subjected to MCAO was reduced in mice treated with GK or edaravone. **(B)** CBF measurements (laser-Doppler flowmetry). **(C-E)** Dose-dependent protective effects of GK on mice brains. Data are expressed as mean ± SD (n = 8). *p < 0.05, **p < 0.01 or ***p < 0.001 vs. MCAO; #p < 0.01 vs. sham. **(F)** Pathological injury visualized by HE staining in mouse brain sections. Scale bar, 50 μm.

To evaluate whether GK is effective in ameliorating cerebral I/R injury, three dosages of GK (2, 4 or 8 mg/kg) were tested in mice subjected to transient middle cerebral artery occlusion (MCAO). Edaravone (10 mg/kg), a neuroprotective free radical scavenger, was used as positive control. Results showed that GK dose-dependently improved brain deficits in brain infarct volume, neurological function, and brain water content (Figure 5A, 5C, 5D, and 5E). Also, Laser-Doppler flowmetry showed that cerebral blood flow (CBF) was restored by GK treatment (Figure 5B). Notably, the benefits of GK at the 8 mg/kg dose were even greater than those observed with edaravone.

Histological evaluation by hematoxylin-eosin (HE) staining was conducted to identify neuronal cell loss in the cortex ipsilateral to MCAO (Figure 5F). HE staining showed that neurons were shrunk and the number of neurons was decreased significantly after MCAO surgery. However, neuronal damage was partially prevented by both GK (8 mg/kg) and edaravone. Collectively, these data demonstrate that GK alleviates brain injury after I/R in an animal model of stroke.

### GK attenuates Drp1 and GSK-3β translocation to mitochondria after I/R injury *in vivo*

**Figure 6:**
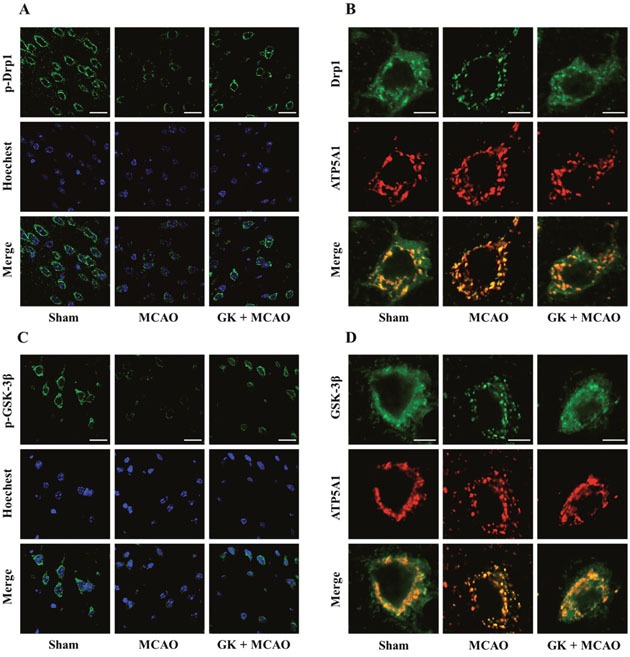
GK attenuates Drp1 and GSK-3β mitochondrial translocation caused by I/R injury in the mouse brain **(A-B)** GK administration inhibited mitochondrial recruitment of Drp1 caused by I/R. Mouse brains were immunostained with p-Drp1 (Ser637) **(A)** or with Drp1 and ATP5A1 **(B)**. **(C-D)** GK treatment attenuated I/R-induced GSK-3β translocation from cytoplasm to mitochondria. Mouse brain sections were immunostained with p-GSK-3β (Ser9) **(C)** or with GSK-3β and ATP5A1 **(D)**.

To validate the effects of GK observed in cultured neuronal cells, immunofluorescence staining was performed in mouse brain slices. p-Drp1 (Ser637) staining showed decreased signal after I/R injury, and this effect was attenuated in brain sections from mice treated with GK (Figure 6A). In addition, double staining against ATP5A1 and Drp1 was used to verify Drp1 localization to mitochondria. Immunofluorescence images showed that I/R-induced mitochondrial translocation of Drp1 was inhibited by GK treatment (Figure 6B).

Mouse brain slices stained with p-GSK-3β showed that phosphorylation of GSK-3β at Ser9 decreased after I/R injury, and GK treatment counteracted such decrease (Figure 6C). Consistent with this result, brain slices double stained with ATP5A1 and GSK-3β confirmed that GSK-3β translocation to mitochondria was also prevented by GK (Figure 6D). Consistent with our *in vitro* data, these findings suggest that GK reduces mitochondrial translocation of Drp1 and GSK-3β after an I/R insult *in vivo*.

## DISCUSSION

Mitochondria generate extensive ROS during AIS, leading to mitochondrial fission and dysfunction, which contribute to characteristic pathological changes in the brain [16, 19]. A previous study showed that GK, a terpene trilactone extracted from the leaves of *Ginkgo biloba*, alleviates cerebral I/R injury through reducing glutamate- or H_2_O_2_-induced cytotoxicity [20, 21]. In this study, we used *in vitro* and *in vivo* models of post-stroke I/R injury to investigate the ability of GK to protect neurons from mitochondrial dysfunction and apoptotic cell death. Our results demonstrated that GK reduced the extent of mitochondrial fragmentation by increasing Drp1 phosphorylation at Ser637 and preventing its translocation to mitochondria. *In vivo* data further confirmed that GK attenuated brain damage through inhibiting mitochondrial Drp1 recruitment.

When mitochondria undergo an ischemic challenge, their morphology and the permeability of their inner and outer membranes are affected, resulting in the opening of transmembrane channels, a collapse in the organelle's membrane potential, and disruption of the respiratory chain with uncoupling of oxidative phosphorylation, all of which contributes to cell energy depletion and ultimately to cell death [11, 22]. Our work showed that GSK-3β inhibition alleviated neuronal damage through regulating mPTP opening, in accordance with a previous study showing that phosphorylation of GSK-3β protected against cardiac reperfusion damage [23]. Our data also showed that GK maintained mitochondrial integrity and inhibited mitochondrial GSK-3β binding after I/R. We further showed that Drp1 and GSK-3β are recruited to mitochondria in mouse brains after MCAO surgery, and GK had protective effects by inhibiting Drp1 and GSK-3β translocation to mitochondria in the infarcted brains.

It was previously shown that the presence of elongated mitochondria is associated with decreased mPTP sensitivity and reduced cell death in ischemic heart injury [24]. Mitochondria-bound Drp1 facilitates Bax oligomerization in the outer mitochondrial membrane, increasing its permeability [18]. Another mitochondrial fission mechanism associated with mPTP opening in I/R injury is illustrated in our study. The mitochondrial fission inhibitor Mdivi-1 increased the phosphorylation of GSK-3β at its Ser9 residue; since phospho-GSK-3β (Ser9) binds to ANT directly, the affinity of ANT toward CypD, the crucial regulator of mPTP, is reduced. This mechanism is in agreement with results from a previous study showing that phospho-GSK-3β (Ser9) increased the threshold of mPTP by reducing the physical interaction of ANT and CypD [13]. GK exerted a similar effect as Mdivi-1, i.e. increased GSK-3β phosphorylation and attenuated CypD-dependent mPTP opening, thus confirming that GK attenuated brain injury after ischemic stroke by maintaining mitochondrial structural and functional integrity. On the other hand, Co-IP results showed that Drp1 binds directly to GSK-3β in N2a cells, in line with previous results in an Alzheimer's disease study [25]. The translocation of GSK-3β to mitochondria was regulated by Drp1 recruitment, and the observed neuroprotective effect may be based on the extent of their interaction. Drp1 and GSK-3β can both be activated by serine/threonine-specific protein kinases like Akt and AMPK, a mechanism to be addressed in a future study.

Prolonged mPTP opening results in MMP depolarization and cytochrome *c* release, followed by caspase-9 and caspase-3 activation-mediated apoptosis. We demonstrate that GK treatment attenuated caspase activation, thus enhancing neuronal survival.

**Figure 7:**
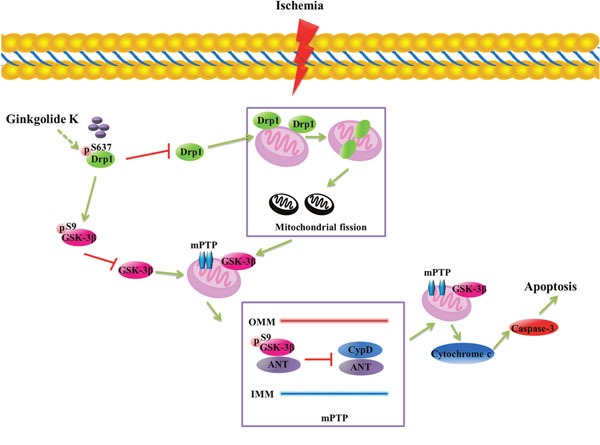
Proposed model of GK's protective actions against mitochondrial dysfunction induced by I/R injury GK inhibits Drp1 activation by inducing Drp1 phosphorylation at Ser637, and attenuates mPTP opening in a GSK-3β-dependent manner. GK treatment also reduces the affinity of ANT for CypD in mitochondria by increasing the binding of p-GSK-3β (Ser9) to ANT, thus preventing cytochrome *c* release and apoptosis.

In summary, we propose that GK has a dual role in neuronal protection after I/R injury by inhibiting mitochondrial fission via phosphorylation of Drp1 (Ser637), and attenuating mPTP opening in a GSK-3β-dependent manner in neurons (Figure 7). Our data provides evidence that Drp1 and GSK-3β may be important factors in mPTP opening after cerebral I/R injury, and GK administration may significantly attenuate AIS sequelae.

## MATERIALS AND METHODS

### Chemicals and reagents

Fetal bovine serum (FBS), trypsin, and Hank's solution were purchased from Gibco (Grand Island, NY, USA). Dulbecco's Modified Eagle's Medium (DMEM), Hoechst 33342, tetramethylrhodamine ethyl ester (TMRE), calcein-AM, and Cyclosporin A (CSA) were brought from KeyGEN Biotech (Nanjing, China). Mito-Tracker Red, Mito-Tracker Green, and Mito-Sox Red were obtained from Invitrogen (Carlsbad, CA, USA). Chir99021 was purchased from Selleck Chemicals (Houston, TX, USA). Edaravone, Mdivi-1, and Mito-TEMPO were from Sigma (St Louis, MO, USA). These reagents were dissolved in dimethyl sulfoxide (DMSO) and the final DMSO concentration was <0.1% v/v. Primary antibodies and secondary antibodies for western blot analyses are listed in Supplementary Tables 1 and 2. Cytochrome *c* ELISA kit was purchased from Cusabio (Wuhan, China) and the intracellular calcium kit was from Beyotime Biotechnology (Nanjing, China).

### Animals

All animal experiments strictly followed the Provisions and General Recommendation of Chinese Experimental Animals Administration Legislation. Male C57BL/6 mice (18-22 g) were purchased from the Comparative Medicine Center of Yangzhou University, China.

### Middle cerebral artery occlusion (MCAO)

Middle cerebral artery occlusion surgery was carried out as reported previously [26, 27]. Mice were deeply anesthetized with 3% chloral hydrate (450 mg/kg) and restrained in the supine position. A midline incision was made in the neck to expose the right common carotid artery (CCA), external carotid artery (ECA), and internal carotid artery (ICA). A 0.18 mm diameter nylon filament (Jialing, 1800) was inserted into the ICA through the ECA stump to block the blood flow to the middle cerebral artery (MCA). The filament was gently advanced into the ICA until mild resistance was felt. Cerebral blood flow (CBF) was measured by a laser-Doppler flowmeter (moorFLPI, Axminster, UK). Mice that attained at least an 80% decrease in cerebral blood flow were included in the study. Animals were kept in a thermostat at 37°C during 1h MCA occlusion. For reperfusion, mice were anesthetized and the filament was carefully withdrawn. After neck incision suturing, mice were returned to their home cages.

### Drug treatments

Ginkgolide K (GK) was kindly provided by Jiangsu Kanion Pharmaceutical Co., Ltd. (China). GK was dissolved fully in normal saline mixed with glycerine, ethanol, and DMSO. Edaravone was prepared by the same method used for GK. Drugs were administrated intraperitoneally at the onset of reperfusion at the following dosages: GK, 2, 4, and 8 mg/kg; edaravone, 10 mg/kg.

### Neurologic deficit score

Twenty-four hours after reperfusion, neurological deficits were measured by a five-point scoring system developed by Longa et al [28], as follows: no neurologic deficit (0); failure to fully extend the left forepaw (1); circling to the left (2); falling to the left (3); falling to the left plus depressed level of consciousness (4).

### Infarction volume analysis

After 24h of reperfusion, mice were sacrificed and their brains were immediately dissected out, sliced, and stained with 2% 2,3,5-triphenyltetrazolium chloride (TTC) for 15 min at 37°C. Then brain sections were fixed in 4% formaldehyde overnight. Slices images were taken with a digital camera (Nikon, Coolpix) and analyzed using image analysis software (Image-Pro Plus, Version 6.0). Infarct volume was measured by the equation:

Infarct volume (%) = Infarct volume/ Total volume of slice × 100%.

### Brain water content assessment

After 24h of reperfusion, brain water content was determined by differential weighing. Brain hemispheres were rapidly dissected, weighed to obtain wet weight, and dried at 105°C for 24h until a constant weight was attained (dry weight). Brain water content was calculated as [(wet weight - dry weight)/wet weight] × 100%.

### Histopathological assessment

At 24h post reperfusion, mice brains were immediately dissected out, fixed with 4% paraformaldehyde overnight, and embedded in paraffin. Brain slices were stained with hematoxylin and eosin (HE) and images recorded using a Nikon Eclipse Ti microscope (Nikon, Japan).

### Oxygen-glucose deprivation/reoxygenation (OGD/R) model

Mouse neuroblastoma N2a cells were purchased from the American Type Culture Collection (ATCC). Cells were maintained in DMEM supplemented with 10% FBS, 100U/mL penicillin, and 100 μg/mL streptomycin, and cultured in a 95% air and 5% CO_2_ atmosphere. Cells were switched to serum-free medium for 2-4h before drug treatments, started 4h before OGD/R. After two washes in glucose-free Earle's buffered salt solution (EBSS), cells were exposed to OGD in an incubation chamber filled with 94% N_2_, 5% CO_2_, and 1% O_2_ for 4h. Cells were then reoxygenated in regular culture medium for 1h. Cells cultured in serum-free medium without OGD/R served as controls. After these treatments, different tests were carried out as described below.

### Mitochondrial reactive oxygen species, mitochondrial fission, mPTP opening, mitochondrial membrane potential, and intracellular calcium assays

For mitochondrial ROS measurements, N2a cells were pretreated with GK (40 μM) or Mito-TEMPO (10 μM) and exposed to OGD/R. Cells were then incubated with MitoSox Red (5 μM) and Mito-Tracker green (300 nM) for 30 min. Stained cells were viewed by confocal microscopy. Several random fields (≥ 50 cells) were evaluated in three independent experiments.

The mitochondrial fission assay was conducted by staining cells with Mito-Tracker Red. Cells were treated with GK (40 μM), Mdivi-1 (5 μM), or Mito-TEMPO (10 μM), and then exposed to OGD/R. Cells were then washed with PBS and stained with Mito-Tracker Red CMXRos (400 nM) for 30 min at 37°C. Confocal microscopy was used to assess mitochondrial morphology. Several random fields (≥ 50 cells) were evaluated in three independent experiments.

Calcein-AM fluorescence was measured using a microplate reader equipped with 485 nm excitation and 510 nm emission filters. After OGD/R, N2a cells were treated with calcein-AM (2.5 μM) for 15 min and then incubated with CoCl_2_ (4 mM) for 15 min.

For detection of mitochondrial membrane potential (MMP), N2a neurons were challenged with OGD/R and incubated with the potentiometric dye TMRE (500 nM) for 30 min at 37°C and with Hoechst 33342 for 10 min. MMP was measured in fluorescence microscopy images from three independent experiments.

For measurement of intracellular calcium, following OGD/R N2a cells were labeled with Fura-2 AM (2 μM) for 30 min. Fura-2 AM fluorescence was detected by a microplate reader at 340 nm/510 nm excitation/emission wavelengths.

### Apoptosis assay

Cells were treated with GK (40 μM), Mdivi-1 (5 μM), or Chir99021 (5 μM) and then exposed to OGD/R. Apoptosis was detected by flow cytometry (FACSCalibur, BD Biosciences, USA) using the AnnexinV-FITC/PI Apoptosis Detection Kit (Becton Dickinson, Rutherford, NJ, USA) according to the manufacturer's instructions.

### Immunoblotting and immunoprecipitation

To prepare total cell protein extracts, cells were lysed in cold radioimmunoprecipitation assay (RIPA) buffer for 30 min. A protease inhibitor cocktail, a phosphatase inhibitor cocktail (PhosStop, Roche, Indianapolis, USA), and PMSF (KeyGEN Biotech, Nanjing, China) were added to RIPA buffer. The cell lysate was centrifuged at 12,000 g, 4°C, for 10 min. After centrifugation, the supernatant was mixed with protein sample buffer and denatured at 95°C for 10 min. Protein concentration was measured by the BCA method following manufacturer's instructions.

To prepare cytosolic and mitochondrial protein samples, cells were collected in mitochondrial isolation reagent (KeyGEN Biotech, Nanjing, China) and homogenized using a glass homogenizer. Cell homogenates were centrifuged at 1,000 g, 4°C, for 10 min. The supernatant was collected and centrifuged at 12,0 00 g, 4°C for 10 min. Cytosolic protein was collected carefully from the supernatant, and mitochondria were recovered from the pellet. Mitochondria were lysed in cold mitochondrial lysate (KeyGEN Biotech, Nanjing, China) to obtain mitochondrial proteins. Equal amounts of proteins were loaded, separated by electrophoresis, and transferred to PVDF membranes by wet electrophoretic transfer. The proteins were incubated with primary antibodies at 4°C overnight and immunoblotted with HRP-conjugated secondary antibodies at room temperature for 3h. Finally, the protein bands were developed by western blotting substrate (Tanon, Shanghai, China) and analyzed with a Tanon-5200 Chemiluminescent imaging system (Tanon, Shanghai, China).

For immunoprecipitation assays, cells were lysed with cold RIPA buffer containing a protease inhibitor cocktail, PhosStop, and PMSF. The cell lysate was centrifuged at 12,000 g, 4°C, for 10 min, and the supernatant was collected and incubated with antibodies for 4h at 4°C. Then the solution was incubated with fresh protein A+G agarose for 4h at 4°C. The agarose was then washed 3 times using 500 μl of PBS and boiled in protein sample buffer at 95°C for 10 min. Equal amounts of proteins were used to perform immunoblot analyses as described above.

### Immunofluorescence staining

Paraffin-embedded mouse brain sections were dewaxed in xylene twice and dehydrated. Antigen retrieval was carried out at 95°C for 15 min in sodium citrate buffer (10 mM sodium citrate monohydrate, pH 6.0). Endogenous peroxidase was blocked by adding 3% H_2_O_2_ in 80% methanol for 10 min. Brain sections were permeabilized with 0.1% Triton X-100 and nonspecific binding sites were saturated with 5% BSA for 1h. Following incubation with primary antibodies for 12h at 4°C, the slides were washed and incubated with FITC-labeled anti-rabbit and Cy3-labeled anti-mouse IgG (H+L) antibodies for 3h at room temperature. After washing, samples were stained with Hoechst 33342 for 10 min at room temperature, mounted, and visualized by confocal microscopy (Zeiss LSM700).

### Cytochrome *c* analysis

After OGD/R challenge, mitochondrial and cytosolic proteins were prepared as described above. An ELISA kit was used for cytochrome *c* quantification according to the manufacturer's instructions (Cusabio Biotech, WH, China).

### Statistical analysis

All the data are expressed as mean ± SD (standard deviation), and values express results from experiments performed in triplicate. Data were analyzed by one-way ANOVA with Dennett's post hoc test. A value of p < 0.05 was considered as significant.

## SUPPLEMENTARY MATERIALS TABLES



## Abbreviations

AIS: acute ischemic stroke
GK: ginkgolide K
I/R: ischemia and reperfusion
mPTP: mitochondrial permeability transition pore
ANT: adenine nucleotide translocator
CypD: cyclophilin D
ROS: reactive oxygen species
MMP: mitochondrial membrane potential
TMRE: tetramethylrhodamine ethyl ester
MCAO: middle cerebral artery occlusion
DMSO: dimethyl sulfoxide
TTC: 2, 3, 5-triphenyltetrazolium chloride monohydrate
EBSS: Earle's buffered salt solution
PMSF: phenylmethanesulfonyl fluoride
