# Supplementary material for: Ginkgolide K attenuates neuronal injury after ischemic stroke by inhibiting mitochondrial fission and GSK-3β-dependent increases in mitochondrial membrane permeability
Source: Oncotarget. 2017 May 18;8(27):44682–93. doi: 10.18632/oncotarget.17967 (PMC5546510; doi:10.18632/oncotarget.17967)
Supplement: Supplementary file 1 [file oncotarget-08-44682-s001.pdf]

Ginkgolide K attenuates neuronal injury after ischemic stroke by inhibiting mitochondrial fission and GSK-3β-dependent increases in mitochondrial membrane permeability

Supplementary Materials

Supplementary Table 1: Primary antibodies used in this study

Name of antibody	Type	Company	Cat no	Application	Dilution
**Phospho-Drp1 (Ser637)**	Rabbit	Abcom	ab193216	WB<br>IF	1:250<br>1:50
**Drp1**	Rabbit	Cell signaling	8750	WB	1:1000
**GSK-3β**	Rabbit	Cell signaling	9315	WB<br>IF	1:1000<br>1:100
**Phospho-GSK3β (Ser9)**	Rabbit	Cell signaling	9323	WB	1:1000
**Cleaved-Caspase-3**	Rabbit	Cell signaling	9664	WB	1:1000
**Cleaved-Caspase-9**	Rabbit	Cell signaling	9501	WB	1:1000
**Bax**	Rabbit	CST	2774	WB	1:1000
**β-actin**	Rabbit	Bioss	bs-0061R	WB	1:1000
**COX-4**	Rabbit	Bioss	bs-1533R	WB	1:1000
**Cyclophilin D**	Rabbit	Bioss	bs- 9878R	IP<br>WB	1:200<br>1:1000
**ANT**	Rabbit	Bioss	bs- 6794R	WB	1:1000
**Drp1**	Rabbit	Bioss	bs- 4100R	IP<br>WB	1:200<br>1:1000
**ATP5A1**	Mouse	Proteintech	60029-1-lg	IF	1:100

IF: immuno-fluorescent staining; IP: immune precipitation; WB: Western blotting.
Rabbit anti-Drp1 antibody (bs-4100R) was used in cytosolic Drp1 analysis and IP assay.
Rabbit anti-Drp1 antibody (8570) was applied in mitochondrial Drp1 expression assay.

Supplementary Table 2: Secondary antibodies used in this study

Name of antibody	Type	Company	Cat no	Application	Dilution
**Anti-mouse lgG (H+L) HRP**	Goat	ZSGB-BIO	ZB-2306	WB	1:1000
**Anti-rabbit lgG (H+L) HRP**	Goat	ZSGB-BIO	ZB-2301	WB	1:1000
**FITC-labeled Anti-rabbit lgG(H+L)**	Goat	Beyotime	A0562	IF	1:100
**Cy3-labeled Anti-mouse lgG(H+L)**	Goat	Beyotime	A0521	IF	1:100

IF: immuno-fluorescent staining; WB: Western blotting.
